# Comparative Study of Laboratory Versus Bedside High-Sensitivity Troponin I in the Emergency Medicine Department of a Tertiary Care Hospital in India

**DOI:** 10.7759/cureus.66512

**Published:** 2024-08-09

**Authors:** Varsha Shinde, Yash Dixit, Pranay Penmetsa, Avinav Luthra

**Affiliations:** 1 Department of Emergency Medicine, Dr. D. Y. Patil Medical College, Hospital and Research Centre, Dr. D. Y. Patil Vidyapeeth (Deemed to be University), Pune, IND; 2 Department of Emergency Medicine, United Institute of Medical Sciences, Prayagraj, IND

**Keywords:** point-of-care test, high-sensitivity troponin i (hstni), acute myocardial infarction, emergency medicine research, non-st segment elevation myocardial infarction (nstemi)

## Abstract

Background: Evaluating high-sensitivity troponin I levels in emergency medicine is critical for diagnosing acute myocardial infarction (AMI). This study aims to evaluate the central laboratory versus bedside troponin I test in the emergency department of a tertiary care center.

Material and methods: This prospective observational study was conducted at Dr. D. Y. Patil Medical College, Hospital and Research Centre, Pune, Maharashtra, India, from October to December 2023. Patient samples were analyzed in the central laboratory using the Dimension EXL 200 (Siemens® Healthcare Diagnostics Inc., Erlangen, Germany) as the gold standard test and through point-of-care testing using the TriageTrue® (Quidel Corporation, San Diego, CA) high-sensitivity troponin I kit, which was run on the Triage® MeterPro® device (Quidel Corporation, San Diego, CA). This device quantitatively determines troponin I in ethylenediaminetetraacetic acid-anticoagulated whole blood and plasma specimens. The results were compared. Statistical analysis was performed using SPSS version 18 (SPSS Inc., Chicago, IL). An unpaired t-test was performed to compare the difference in time taken using the two testing methods.

Result: The mean time for obtaining troponin I results was substantially shorter with bedside testing (14.91 minutes, standard deviation (SD) = 0.5) than with laboratory testing (119.1 minutes, SD = 5.03). Statistical analysis revealed a significant difference (t = -172.36, p < 0.001). A chi-square test was conducted to assess the disparity between the two testing methods, yielding a chi-square value of 32.64 and a p value of 0.00001, indicating a significant difference between bedside testing and laboratory testing.

Conclusion: The bedside high-sensitivity troponin I test offers a considerable advantage over laboratory testing regarding turnaround time within the emergency medicine department in India. This rapid diagnostic capability is crucial for timely management, which is beneficial for patients inconclusive of acute coronary syndrome-like non-ST segment elevation myocardial infarction (NSTEMI). It is also cost-effective. It also reduces the emergency boarding time and may reduce the number of unnecessary admissions in healthcare facilities.

## Introduction

Chest pain is a frequent reason for people to visit the emergency room worldwide [1]. The differentials for chest pain range from minor issues like muscle pain to serious conditions like a heart attack [2]. Developing nations bear a larger portion of the cardiovascular disease (CVD) burden compared to developed ones. CVD takes nearly as many lives as diabetes, cancer, and accidents combined [3]. An electrocardiogram (ECG) is used to differentiate between ST-elevation myocardial infarction (MI) and non-ST elevation MI (NSTEMI). Meanwhile, cardiac troponin levels are instrumental in distinguishing between NSTEMI and unstable angina UA, especially when the ECG findings are nonspecific or normal.

Acute coronary syndrome (ACS) patients are considered triage category red in the emergency department (ED). Therefore, the management and prioritization of these patients are of utmost importance. So, early diagnosis and treatment can lower mortality rates. ED physicians must assess the risk, prioritize patients, and promptly initiate treatment for cases of chest pain. In a heart attack (MI), a blocked blood vessel deprives oxygen to part of the heart, causing it to die. Without timely treatment, the heart muscle can be irreversibly damaged. Identifying myocardial necrosis postmortem typically takes about six hours [4]. The key to effective treatment is promptly diagnosing MI to initiate treatment as soon as possible. Cardiac markers, such as troponin I, creatine kinase-MB, or troponin T (TNT), play a crucial role in identifying ACS when ECG changes are absent in patients presenting with chest pain. In as many as 50% of cases with inconclusive ECG readings, troponin tests are pivotal in diagnosing acute MI (AMI) [5]. However, diagnosing ACS is often delayed because these markers are typically analyzed in a central laboratory outside the ED. The measurement of cardiac markers is essential in assessing patients with chest pain. Yet, many hospitals still struggle to meet the turnaround time from blood collection to final results reporting [6].

Patient care has changed significantly since the advent of bedside tests, sometimes called point-of-care (POC) tests, when blood tests are performed close to the patient's location, especially in emergency rooms. This advancement has sped up the evaluation and decision-making process for patients in the ED by considerably reducing the waiting times for radiological exams and laboratory test results. For patients suspected of having a heart attack, POC testing has decreased result waiting times by approximately one hour, reduced ED length of stay by two hours, and shortened the time to treatment [7-10].

This comparative study assesses the efficacy and feasibility of central laboratory-based versus bedside troponin I testing in the Emergency Medicine Department. It intends to evaluate the diagnostic accuracy, turnaround time, and clinical utility of both methods in diagnosing AMI and optimizing patient management. By avoiding unnecessary admissions to the ED, the study will also save money.

## Materials and methods

Study design and setting

This was a prospective observational study. This study was conducted in the Emergency Medicine Department at Dr. D. Y. Patil Medical College, Hospital and Research Centre, Pimpri, Pune, over three months from October 2023 to December 2023. The study aimed to assess the effectiveness of laboratory-based versus bedside high-sensitivity troponin I tests in diagnosing ACS among patients presenting with chest pain. Data analysis was conducted, considering the limited number of bedside troponin I strips provided for the study. Central laboratory-based and bedside troponin I tests were conducted as part of the investigation to compare their diagnostic accuracy and clinical utility within the context of the Emergency Medicine Department.

Ethical consideration

The procedures adhered to the ethical standards set by the Ethics Review Committee at Dr. D. Y. Patil Medical College, Hospital and Research Centre. Patient obscurity was maintained by avoiding the use of names or patient registered numbers in both text and illustrations. The study received approval from the ethical committee, and patient confidentiality was rigorously upheld by the ethical committee. All data input forms were assigned and made accessible only to authorized personnel. Patients provided consent before participating in the study.

Participant selection

The study included patients aged 18 years or above who presented with chest pain with no typical ECG changes and who provided consent for participation. Patients below 18 years of age, with ST elevation in ECG, and those who did not consent to participate were excluded.

Sample size

**Figure 1:**
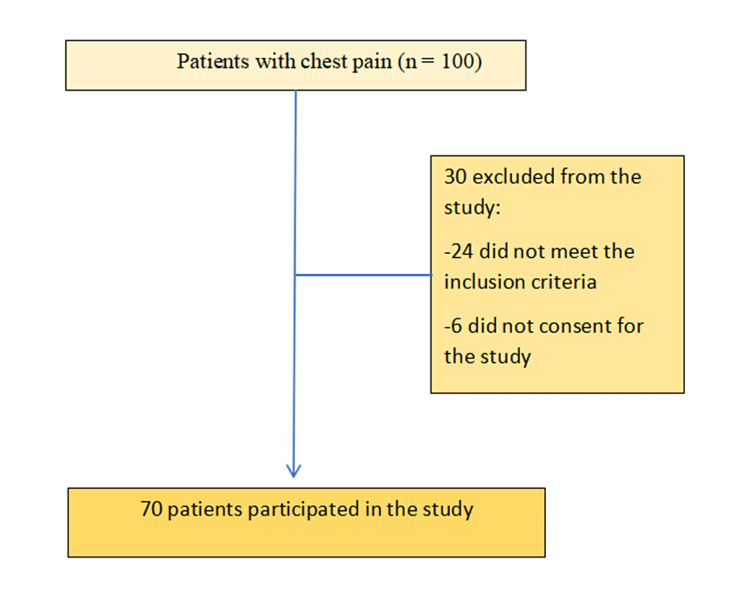
Flow diagram showing the patient selection

A total of 100 patients presented with chest pain and were considered for enrollment, of which 30 were excluded from the study due to lack of consent or typical ST-elevation findings on ECG, as shown in Figure 1. The study encompassed 70 participants admitted to Dr. D. Y. Patil Medical College, Hospital and Research Centre, Pimpri, Pune.

Methods of data collection

Selected patients were those who arrived at the emergency room with complaints of chest pain along with signs and symptoms of NSTEMI or ACS. Once consent was obtained, the TriageTrue® (Quidel Corporation, San Diego, CA) high-sensitivity troponin I bedside test was performed on patients. Due to its sensitivity to heat and humidity, the test kit was kept between 2°C and 8°C in storage. After taking the kit out of its box, 175 μL of whole blood or plasma sample was added using a transfer pipette to begin the test. It took 15 minutes to get the test results. As a gold standard test ran in the Dimension EXL 200 (Siemens® Healthcare Diagnostics Inc., Erlangen, Germany) for high-sensitivity troponin I, one sample was submitted concurrently to the hospital's central laboratory.

Statistical analysis

Descriptive statistics, including means, proportions, and frequencies, were used to present the data. The specificity and sensitivity kit was assessed against the gold standard test, central laboratory Dimension EXL 200, which runs high-sensitivity troponin I. Statistical analysis was performed using SPSS version 18 (SPSS Inc., Chicago, IL).

Procedure

The high-sensitivity troponin I test is a one-time use fluorescence immunoassay tool to measure high-sensitivity troponin I levels in plasma or whole blood specimens anticoagulated with ethylenediaminetetraacetic acid. The test technique adds 175 μL of whole blood or plasma to the TriageTrue® test device's sample port. A filter built into the TriageTrue® device is used to separate the plasma from the whole blood cells in specimens that contain whole blood. The test kit is placed inside the Triage® MeterPro® (Quidel Corporation, San Diego, CA) apparatus. After reacting with fluorescent antibody conjugates, the sample enters the test apparatus through capillary action. Complexes of the fluorescent antibody conjugate are formed on designated zones specific for high-sensitivity troponin I within 15 minutes.

The central laboratory high-sensitivity troponin I assay is run in Dimension EXL 200, a two-step chemiluminescent microparticle immunoassay for the quantitative determination of high-sensitivity cardiac troponin I with a flexible assay protocol referred to as Chemiflex. The central laboratory high-sensitivity troponin I study is considered the gold standard test.

Measurement tools

In this study, the TriageTrue® high-sensitivity troponin I test kit and Triage® Meter Pro® were utilized for bedside testing. The central laboratory conducted the gold standard test on the Siemens® Dimension EXL 200, revealing a cutoff value of 20.5 ng/L for high-sensitivity troponin I.

## Results

These results illustrate the distribution of participants across different age groups. Most participants were 30-50 years old, comprising 44.3% of the total sample. Participants aged less than 30 accounted for 20% of the sample, whereas those aged between 50 and 70 constituted 30% of the total. Only a small percentage of participants (5.7%) were over 70 years old. These findings provide valuable demographic insights into the study population, which are essential for understanding the characteristics of the patients included in the research (Table 1).

**Table 1:** Demographics of the study population

Age group	Frequency (n)	Percentage (%)
<30 years	14	20%
>30 to 50 years	31	44.3%
>50 to 70 years	21	30%
>70 years	4	5.7%
Total	70	100%

These results depict the distribution of participants by gender (Table 2). Most participants were male, comprising 67.1% of the total sample, whereas females accounted for 32.9% of the participants.

**Table 2:** Gender distribution of the population

Gender	Frequency (n)	Percentage (%)
Male	47	67.1%
Female	23	32.9%
Total	70	100%

A chi-square test was conducted to assess the disparity between the two testing methods, as shown in Table 3, showing a true positive of n = 24, false negative of n = 14, false positive of n = 28, and true negative of n = 4, yielding a chi-square value of 32.64 and a p value of 0.00001, indicating a significant difference between bedside testing and laboratory testing (p < 0.01).

**Table 3:** Contingency table ^a^True positives (gold standard positive and new bedside test positive) ^b^False negatives (gold standard positive and new bedside test negative) ^c^False positives (gold standard negative and new bedside test positive) ^d^True negatives (gold standard negative and new bedside test negative)

Test	Bedside positive	Bedside negative	Total (n)
Gold standard positive	33^a^	5^b^	38
Gold standard negative	6^c^	26^d^	32
Total	39	31	70

The positive predictive value is 84.6%, and the negative predictive value is 83.9%. The sensitivity is 86.8%, and specificity is 81.25%. These findings provide valuable insights into the diagnostic accuracy and performance of high-sensitivity troponin I testing conducted at the bedside compared to central laboratory testing. The observed significant difference underscores the importance of considering the testing method in clinical decision-making. In bedside testing, the mean time for obtaining high-sensitivity troponin I results was 14.91 minutes, with a standard deviation (SD) of 0.5 minutes. In contrast, for laboratory testing, the mean time taken was considerably longer at 119.1 minutes, with an SD of 5.03 minutes. An unpaired t-test was performed to assess the difference in time taken between the two testing methods, resulting in a t-value of -172.36 and a p-value of less than 0.001, indicating a highly statistically significant difference.

## Discussion

Early diagnostic testing in the ED is essential for identifying patients presenting with chest pain. Given the rapid turnaround time of POC analysis, ensuring the validity of its data is critical. This study's findings, consistent with prior research, demonstrate that the POC system is a reliable test for promptly assessing patients in the ED with symptoms suggestive of ACS [11,12].

Bedside testing revealed positive troponin I levels in 33 participants, compared to 39 participants in laboratory testing, as the chi-square test showed a significant difference (p = 0.00001). The sensitivity, specificity, positive predictive value, and negative predictive value of bedside testing were 86.8%, 81.25%, 84.6%, and 83.9%, respectively. These findings highlight differences in diagnostic accuracy between bedside and central laboratory testing, which are crucial for clinical decisions. Bedside testing for high-sensitivity troponin I yielded a mean time of 14.91 minutes (SD = 0.5), whereas laboratory testing took significantly longer at 119.1 minutes (SD = 5.03). An unpaired t-test showed a highly significant difference (t = -172.36, p < 0.001). These results emphasize the considerable time gap between bedside and laboratory testing, with bedside testing offering a rapid diagnostic advantage in emergencies.

Wilke et al. studied the diagnostic accuracy of POC troponin I, POC TNT, and central laboratory TNT, considering renal function in predicting MI [13]. This study highlights the significance of integrating POC cardiac troponin testing into clinical practice. By offering comparable diagnostic performance to central laboratory tests, along with the added benefits of cost savings and rapid result availability, POC tests represent a valuable tool for the early diagnosis and management of MI, particularly in settings where timely access to central laboratory testing may be limited.

A study conducted at University Hospital Hamburg-Eppendorf in Hamburg revealed that employing a single troponin I assay upon admission for patients presenting with chest pain significantly enhanced the precision of early MI diagnosis compared to conventional TNT assays and other cardiac markers [14]. The findings suggest that incorporating troponin I assays into admission protocols for patients presenting with chest pain could enhance diagnostic accuracy and efficiency. The ability to obtain precise results quickly upon admission aids in the rapid clinical decision-making process.

A similar study was conducted at the Karachi Institute of Heart Diseases, Karachi [15], which concluded that the early use of troponin I is beneficial for diagnosing AMI and is also cost-effective. The findings of this study can significantly inform the implementation of bedside high-sensitivity troponin I test protocols in emergency medicine departments. This advancement can reduce diagnostic turnaround time and enhance the timely management of acute cardiac conditions, improving patient outcomes and more efficient resource utilization. Moreover, in peripheral setups and remote areas where central laboratories are not readily accessible, POC testing can be vital in managing suspected AMI. This approach reduces the costs associated with outsourcing samples to central laboratories and facilitates timely and cost-effective referrals of patients presenting with chest pain from peripheral to tertiary health centers. By implementing these protocols, emergency medical services can be enhanced, ensuring that critical cardiac conditions are promptly and accurately diagnosed and treated, regardless of location. This will ultimately improve patient care and optimize healthcare resource allocation.

One of the strengths of our study was that it was carried out in a tertiary healthcare center with a central laboratory and a 24-hour working cardiac care unit. This setup ensured that patients with positive troponin results could be swiftly transferred to the catheterization laboratory for further evaluation and treatment. Additionally, the bedside test kit required less setup and staff training, making it a practical and efficient tool for early diagnosis and management of MI.

Limitations

The primary limitations of this study are its single-center design and relatively small sample size. A study with a larger sample size would enhance the precision of our findings. Future research with more extensive data could further validate and strengthen our results.

## Conclusions

The study comparing laboratory versus bedside high-sensitivity troponin I testing found a significant disparity in time, considerably quicker than the central laboratory testing. This highlights the advantage of bedside testing in providing rapid diagnostic information in emergency scenarios. It is also beneficial in peripheral health setups as it is cost-effective and time-saving. Implementing bedside testing can streamline the management of AMI, especially in areas without easy access to central laboratories. Further multicenter trials with larger sample sizes are recommended to confirm the broader applicability of bedside testing in various healthcare settings.
